# Predicting outcome from dengue

**DOI:** 10.1186/s12916-014-0147-9

**Published:** 2014-09-04

**Authors:** Sophie Yacoub, Bridget Wills

**Affiliations:** Oxford University Clinical Research Unit, Wellcome Trust Major Overseas Programme, Hospital for Tropical Diseases, 764 Vo Van Kiet Street, Ho Chi Minh City, Vietnam; Department of Medicine, Imperial College, Hammersmith Campus, London, UK; Nuffield Department of Clinical Medicine, Centre for Tropical Medicine, Oxford, UK

**Keywords:** Dengue, Severity, Outcome, Risk factors, Biomarkers, Vascular

## Abstract

Dengue is emerging as one of the most abundant vector-borne disease globally. Although the majority of infections are asymptomatic or result in only a brief systemic viral illness, a small proportion of patients develop potentially fatal complications. These severe manifestations, including a unique plasma leakage syndrome, a coagulopathy sometimes accompanied by bleeding, and organ impairment, occur relatively late in the disease course, presenting a window of opportunity to identify the group of patients likely to progress to these complications. However, as yet, differentiating this group from the thousands of milder cases seen each day during outbreaks remains challenging, and simple and inexpensive strategies are urgently needed in order to improve case management and to facilitate appropriate use of limited resources. This review will cover the current understanding of the risk factors associated with poor outcome in dengue. We focus particularly on the clinical features of the disease and on conventional investigations that are usually accessible in mid-level healthcare facilities in endemic areas, and then discuss a variety of viral, immunological and vascular biomarkers that have the potential to improve risk prediction. We conclude with a description of several novel methods of assessing vascular function and intravascular volume status non-invasively.

## Introduction

Dengue is the most important arboviral infection affecting humans, and presents a major challenge for public health services worldwide. Infection can be caused by any of four dengue virus serotypes (DENV1 to 4), transmitted by *Aedes* mosquitoes. Over half the world’s population is thought to live in areas at risk for transmission, and recent estimates suggest that around 400 million infections occur annually, of which 100 million are clinically apparent [1]. The clinical phenotype varies from a mild self-limiting febrile illness through to severe and occasionally life-threatening disease. Symptomatic disease typically follows three phases: an initial febrile phase lasting 3 to 7 days; a critical phase around defervescence, during which complications appear in a small proportion of patients; and a spontaneous recovery phase. Complications primarily affect the vascular system, and include an unusual plasma leakage syndrome that may result in hypovolaemic shock – the potentially fatal dengue shock syndrome (DSS); a coagulopathy sometimes accompanied by bleeding; and organ impairment [2]. Because these severe manifestations occur relatively late in the disease course, often when the infecting virus is no longer detectable in plasma, immune-mediated mechanisms are postulated to play a significant role in pathogenesis [3]. Currently no vaccine is available, and neither antiviral drugs nor immunomodulatory agents have been shown to be effective in reducing morbidity or improving disease outcome [4–7]. However, with good supportive care (primarily judicious use of parenteral fluid therapy to offset plasma volume losses due to leakage), mortality rates of less than 1% are possible even among DSS cases [8,9].

**Figure 1:**
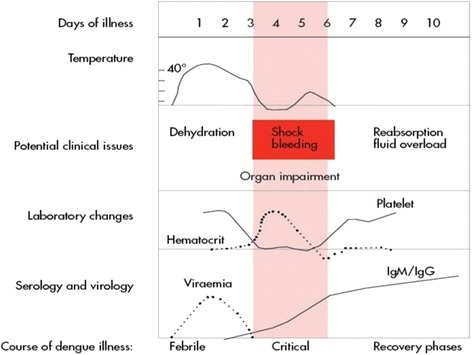
**Dengue disease phases and potential complications.** Reproduced, with permission of the publisher, from the WHO publication [12] (Figure 2.1; p. 25).

Although the vast majority of symptomatic infections do not progress to severe disease, areas of high dengue transmission can have seasonal epidemics, which can quickly overwhelm health service capacity. Thus, the ability to identify patients at high risk of progression, who are likely to benefit from close observation and early intervention with supportive therapy, has become the focus of intense research efforts in recent years. In 2009, the World Health Organization (WHO) revised the classification system for dengue, defining two major entities – dengue and severe dengue – to replace the more complicated dengue fever/dengue haemorrhagic fever (DF/DHF) system used previously [10]. The new classification also encompasses a set of ‘warning signs’ intended to help clinicians identify patients likely to develop complications during the critical phase of the illness (Figure 1). These signs and symptoms were derived in part from a dataset describing almost 2,000 patients with dengue across Asia and Latin America, but owing to the small number of patients progressing to severe disease during the study, further work is needed to validate the findings [11].

This review will focus on the current knowledge of factors associated with disease progression, either considered in terms of DHF/DSS or dengue with warning signs/severe dengue. We concentrate first on readily accessible parameters, that is, the clinical features of the disease and the conventional investigations available in most mid-level healthcare facilities, before discussing a variety of potential viral, immunological and vascular biomarkers that have been proposed. Finally we conclude by describing a number of novel non-invasive methods of assessing vascular function or intravascular volume status, and consider their potential relevance for dengue.

### Clinical features and standard laboratory or radiological investigations

Epidemiological studies have identified a number of risk factors for more severe dengue disease, including secondary infection [13], the two extremes of age [14,15], pregnancy [16], under-nutrition and over-nutrition [17], and the presence of comorbidities such as diabetes and hypertension in adults [18]. Male patients are often overrepresented in dengue epidemics, but female sex has been associated with more severe disease [19–21]. Several groups have attempted to identify the clinical symptoms and signs occurring during the acute illness that are associated with outcome. In a prospective study of 700 Vietnamese children with confirmed dengue, gastrointestinal symptoms and hepatomegaly were more prominent among those classified as having DHF, and a peak haematocrit above 50% was associated with DSS [22]. Clinical bleeding manifestations are highly variable, although skin petechiae are commonly observed across the severity spectrum, and the tourniquet test, one of the original diagnostic criteria for DHF, has been shown to differentiate poorly between DF and DHF [23,24]. In Singapore, where dengue is predominantly an adult disease, a decision tree involving clinical bleeding, high serum urea, low serum protein and low lymphocyte proportion was developed to predict likely progression to DHF among hospitalised patients; sensitivity and negative predictive value were high, but specificity was poor [25]. The decision tree was subsequently validated in another Singaporean cohort, and has been suggested as a tool to guide triage of patients who require hospitalisation versus outpatient treatment [26].

A number of haematological parameters have been considered as potential predictors, most commonly the platelet count [27]. Platelet counts tend to fall over the illness course, reaching a nadir shortly after defervescence, before demonstrating a rapid recovery response [28]. Lower counts (<50× 10^9^/l; normal range 150 to 400 × 10^9^/l) are seen more frequently in severe disease [29], and are considered a risk factor for bleeding [30]. However, the correlation between thrombocytopenia and haemorrhage is weak, with lower platelet counts correlating more closely with the severity of vascular leakage in one study [31].

Although a rapid decrease in platelet count concurrent with an increase in haematocrit is presented as one of the warning signs for likely progression to shock in the 2009 WHO dengue classification [32], specific values have not been defined as yet. Given the wide range of normal platelet values and the fact that the nadir occurs relatively late in the disease course, efforts have been made to develop prognostic algorithms that combine the platelet count with other haematological indices during the febrile phase. Using classification and regression tree analysis, algorithms based on platelet count, total white cell count, monocyte percentage and haematocrit level, obtained within 72 hours of fever onset, identified Thai children who went on to develop DSS with good sensitivity (97%) but limited specificity (48%) [33]. The rate of change in serial haematological markers, particularly the platelet count, may also carry important prognostic information, and efforts are in progress to develop models that incorporate longitudinal data from repeated sampling to see if this improves the accuracy of risk prediction.

Currently, clinical identification of vascular leakage is difficult until or unless DSS develops. The most common method of monitoring leakage relies on identification of relative haemoconcentration, determined by tracking changes in serial haematocrit measurements, with a rise of more than 20% from baseline considered evidence of significant leakage. However, this method can be rather insensitive, particularly if the patient is receiving parenteral fluid therapy, and it is also limited by the fact that an individual’s baseline value is rarely known. Thus, although assessment of haemoconcentration can be helpful for case management, it is often only possible to appreciate the true severity of haemoconcentration once the acute illness has resolved and the haematocrit has returned to normal for that individual. Although comparison with age- and sex-matched population haematocrit values is recommended, such values are rarely available in dengue endemic areas [11].

Hepatic involvement is common in dengue, and liver enzymes are frequently elevated in infections of all severity grades. More marked derangements are usually associated with more severe disease profiles [34,35], but discrimination between severe and non-severe dengue has proved to be poor [36]. In addition, liver enzyme levels tend to peak late in the disease course, typically during the second week, limiting their usefulness as prognostic markers [35]. Hypoproteinaemia is well recognised during the critical phase, and correlates with the severity of leakage [37,38]; however, like haemoconcentration, hypoproteinaemia is difficult to identify without serial measurements or a known baseline value for an individual. Proteinuria also occurs, and marked increases in fractional clearances of several endogenous proteins have been documented among children with DSS [37]. The urine protein/creatinine ratio (UPCR) has been suggested as a possible predictor of outcome, with higher peak UPCRs observed in adult DHF cases compared to DF cases [38]. However, a study describing the magnitude and kinetics of urinary albumin excretion in a large paediatric population found that although albumin excretion was increased in the confirmed dengue patients, with a significant time-trend showing peak values during the critical phase, discrimination between patients of different severity was poor, and albuminuria was not useful in predicting the development of complications [39].

Studies using ultrasound have demonstrated that pleural effusions, ascites and gall bladder wall oedema are common during the critical phase, and correlate with disease severity [40–42]. In addition, serial ultrasound studies indicate that subclinical plasma leakage can be detected as early as days 2 to 3 of fever [43], and is better at predicting likely disease progression than other markers of plasma leakage such as haematocrit and albumin measurements [44]. Gallbladder wall oedema appears to precede the development of ascites and effusions, and may therefore be a helpful early predictor of outcome. Thus ultrasonography can be a useful monitoring tool, and where available, should be considered in the overall assessment during the febrile phase. However, there are certain limitations,particularly the lack of defined normal ranges for the parameters of interest, the variability in measurements obtained by different operators, and the lack of specificity of the findings. For example, gallbladder wall thickness may be increased in other infections and also in the postprandial state [45]. Finally, as yet, the availability of portable ultrasonography and the technical expertise necessary to interpret the images obtained remains relatively restricted in the resource-limited areas where dengue is endemic.

### Potential prognostic biomarkers

In the face of the rapidly expanding global pandemic, many research groups are attempting to assess a variety of potential biomarkers for associations with dengue disease progression, and only a selected overview is possible here. Only human studies will be reviewed, as the true relevance of even the most sophisticated model systems to human dengue disease remains uncertain.

#### Viral and immunological markers

The observations that a) severe disease is more common in secondary infections with a different dengue virus (DENV) serotype and b) severe clinical manifestations typically occur when the viral load is in steep decline [46,47], support the idea that the pathogenesis of severe dengue is at least partly immune-mediated. Antibody-dependent enhancement, whereby cross-reactive antibodies from a previous infection fail to neutralise the current serotype, but instead enhance viral uptake into susceptible cells, is thought to result in a higher blood and/or tissue viral burden and a more intense immune response with increased release of vasoactive mediators [48,49]. Higher viraemias, considered to reflect a greater dengue-infected cell mass, have been linked to severe disease [49,50], with early peak viral loads appearing to correlate with subsequent disease severity. However, serotype differences are apparent, with patients infected with DENV1 sometimes displaying extremely high plasma viraemia levels with relatively mild clinical disease [51,52]. The duration of viraemia has not shown a clear association with disease severity. In fact, rapid viral clearance, represented by the steepness of the decline in viraemia, has been demonstrated more commonly in DHF cases and in secondary infections, possibly as a result of a primed adaptive immune response [50]. Soluble non-structural protein-1 (NS1) levels generally correlate with viraemia levels, and are usually higher in patients with severe than in those with uncomplicated dengue. Higher levels of NS1 within 72 hours of fever onset predicted patients who developed DHF at defervescence in one study [53], and it has been suggested that NS1 may play a pathogenic role in the capillary leakage process, through activation of complement and generation of anaphylatoxins and the terminal complement complex SC5b-9 [54]. Plasma levels of NS1 and SC5b-9 have been shown to correlate with disease severity, and were present in the pleural fluid of patients with DSS, along with the anaphylatoxin C5a. Cross-reactive NS1 antibodies have been shown *invitro* to target membrane-bound NS1, resulting in complement-mediated cytolysis and endothelial cell disruption [55,56]. However, the kinetics of NS1 antigenaemiaare known to be influenced by several factors, including the infecting serotype, with lower levels seen in DENV2 than in DENV1 infections after adjusting for viral load, and the host immunity, with faster clearance times seen in secondary than in primary infections. Thus, the magnitude of NS1 antigenaemia alone is not sufficient to explain outcome, and this would probably affect the overall utility of NS1 as a potential biomarker [52,57].

Numerous studies have investigated associations between altered levels of circulating cytokines/chemokines and complement activation markers with dengue severity. The findings from a selection of representative studies are presented in Table 1 [58–68]. However, interpretation of the data is difficult as most of the studies are small, and confounded by serotype variations and differences in plasma viraemia levels, the age and immune status of the patients, and the timing of sampling during the evolution of the infection. In addition, it is possible that the parameters of interest are influenced by the degree of capillary leakage, making comparisons of plasma measurements between severity groups unreliable.

**Table 1:** **Immunological parameters as potential biomarkers**

**Immune marker**	**Disease phase**	**Comment**
Elevated levels		
IL-4, IL-6, IL-8, IL-10	Elevated during the febrile phase, peaking around defervescence	Elevated levels in patients with DSS compared with those with DF, which correlated with markers of disease severity [58]. IL-10 correlated with degree of thrombocytopenia [49,65]
TNFa, sTNFR-75, sTNFR-80	Elevated in the febrile phase	Raised levels in severe dengue versus mild disease. Positive correlation with disease severity. sTNFRspredicted children who subsequently went on to develop shock [64,67]
IFN-γ	Raised in early febrile phase, peaking prior to defervescence day	DHF was associated with earlier peak IFN-γ levels [49]. Associated with development of severe disease [59]
MIP-1b, G-CSF, IP-10, MCP-1	Elevated in early febrile phase through to defervescence	Compared with healthy control, MIP-1b and G-CSF were elevated in patients with uncomplicated dengue; MCP-1 in dengue with warning signs; and IP-10 in both groups [62]
C3a, C4a, C5a,Factor D	Elevated in acute phase	Elevated in DHF compared with DF [68]
SC5b-9 terminal complement complex	Elevated in acute disease ,peaking day after defervescence	Higher levels were demonstrated in DF/DHF compared with OFI, and correlated with dengue severity [54]
Reduced levels		
RANTES/CCL5	Reduced levels during the acute phase	Reduced during acute phase in patients with dengue compared with healthy controls, and correlated with thrombocytopenia [66]
IL1-b, IL-2, EGF	Reduced levels during febrile phase	No difference was demonstrated between the severity grades for IL-1b but there was significantly lower levels of IL-2 and EGF in DSS compared with DF [58]
VEGF, VEGFR2	Altered levels of VEGF and VEGFR2 around day of defervescence	There are mixed reports of VEGF levels
		One study demonstrated reduced VEGF levels in DSS compared with DF from 2 days before to 2 days after defervescence [58]. Other studies have shown elevated VEGF levels in DHF compared with DF[69], and a further study demonstrated elevated levels of free VEGF in DHF at the time of plasma leakage, and reduced levels of VEGFR2 [63]
C3, factor H	Reduced levels during acute dengue	Reduced levels were found in patients with DHF compared with those with DF and healthy controls [68]

C, complement; DF, dengue fever; DHF, dengue haemorrhagic fever; DSS, dengue shock syndrome;EGF, epidermal growth factor; G-CSF, granulocyte-colony stimulating factor; IFN, interferon; IL, interleukin; IP, interferon-γ-induced protein; MCP, monocyte chemoattractant protein; MIP, macrophage inflammatory protein; OFI, other febrile illness; RANTES/CCL5, regulated on activation, normal T cell expressed and secreted/chemokine (C-C motif) ligand 5; TNF, tumour necrosis factor; sTNFR, soluble tumour necrosis factor receptor; VEGF, vascular endothelial growth factor; VEGFR, vascular endothelial growth factor receptor.

#### Markers of endothelial activation or microvascular disruption

As the vascular system is targeted in dengue infections, there may be a stronger biological rationale for investigating markers specifically implicated in vascular pathology. Soluble intercellular adhesion molecule-1 (sICAM-1) and soluble vascular cell adhesion molecule-1 (sVCAM-1) have been shown to be increased in patients with dengue compared with controls, and to correlate with disease severity [70]. Similarly, sVCAM-1, sICAM-1 and E-selectin were raised in patients with dengue compared with patients with other febrile illness 1 day prior to defervescence [71]. Soluble thrombomodulin, a non-specific marker of endothelial activation, was also found to be increased 2 to 3 days before defervescence in that study, and to correlate well with disease severity [71]. Angiopoietin-2 is a protein secreted from endothelial cells in response to inflammatory stimuli, which antagonises angiopoietin-1, a protein required to maintain effective microvascular barrier function. In one study, reduced levels of angiopoietin-1 and increased levels of angiopoietin-2 were demonstrated in dengue cases at hospital admission compared with levels obtained at discharge and also compared with levels seen in healthy controls, and these changes correlated with plasma leakage and disease severity [72].

In addition to markers of endothelial activation, a few studies have examined factors more directly indicative of disruption of the endothelial barrier. Although morphological examination has revealed only minor non-specific changes in microvascular structure, increased circulating endothelial cells (CECs) have been demonstrated in patients with dengue, with the levels correlating with severity [70,71]. However, even if confirmed to be useful for risk prediction, the techniques required to measure CECs are complex and assay standardisation is difficult, making it unlikely that CEC measurement could be widely deployed in endemic areas. Damage to the endothelial surface glycocalyx layer, a negatively charged gel-like structure that lines the luminal surface of the microvasculature and constitutes the primary permeability barrier, has also been implicated in the pathogenesis of severe dengue. Both the DENV envelope protein and NS1 are known to bind to heparan sulphate, a major constituent of the glyocalyx layer, potentially resulting in shedding of this layer, with consequent alterations in microvascular permeability [73,74]. Preliminary evidence suggests that circulating heparan sulphate levels are raised during the early febrile phase of dengue [75], although further studies are required to determine whether these early increases in heparan sulphate levels predict which patients are likely to develop severe disease.

Along similar lines, microparticles are submicron vesicles that are shed from the surface of cell membranes in response to activation, injury and apoptosis. It is becoming increasingly apparent that far from being simple markers of cell activation, microparticles play a critical role in the pathophysiology of various disease states. Endothelial microparticles are emerging as potential biomarkers for cardiovascular diseases, diabetes and sepsis, conditions in which vascular inflammation and injury are key to patient outcomes [76–78]. Currently, the role of endothelial microparticles in the pathogenesis of the microvascular dysfunction in dengue is not known. However in one study, platelet derived microparticles(PMPs) rich in IL-1b correlated with signs of vascular permeability in patients with dengue, and PMPs were found to induce vascular permeability *invitro* in an IL1-dependent manner [79].

### Novel methods of assessing vascular status clinically

As outlined earlier, the defining feature of severe dengue is disruption of vascular barrier function, resulting in plasma leakage, intravascular volume depletion, and eventually cardiovascular collapse and death if appropriate fluid therapy is not instituted promptly. Current techniques to identify and monitor leakage rely on surrogate markers of intravascular volume depletion, including serial haematocrit measurements, and close observation of cardiovascular indices, particularly the pulse pressure and heart rate [10]. However, these parameters are relatively insensitive, and show little change until the critical phase of the infection (Figure 1), whereas evidence from serial ultrasound studies indicates that plasma leakage actually starts during the febrile phase [44]. It is possible that many patients experience some degree of plasma leakage, and that only a minority become haemodynamically unstable when a significant volume of fluid has extravasated, overwhelming that individual’s intrinsic compensatory homeostatic mechanisms. Several new techniques designed to evaluate endothelial dysfunction, monitor the microcirculation, and assess intravascular volume status have been developed in recent years, and have the potential to be useful tools for predicting outcome in dengue.

#### Evaluating endothelial dysfunction

Greater understanding of normal microvascular physiology together with increasing recognition of the importance of endothelial dysfunction in systemic disease has led to the development of endothelial function testing in a variety of medical disciplines. Available techniques include venous plethysmography, assessment of flow-mediated dilatation of the brachial artery and peripheral artery tonometry (PAT). The latter two techniques evaluate endothelial function through monitoring the reactive hyperaemia (RH) response following occlusion of a proximal artery.

In PAT, changes in amplitude of the digital pulse volume are postulated to reflect nitric oxide bioavailability. The increase in blood flow following an occlusion test is thought to stimulate production and release of endothelial-derived nitric oxide, causing vasodilation. The test is easy to apply, requires less staff training and has better inter-user variability than techniques to assess brachial artery dilatation, and has shown good reproducibility [80,81].

Longitudinal studies involving serial RH assessments have demonstrated that altered endothelial responses can be identified several years prior to the occurrence of adverse cardiovascular events [82,83]. More recently, endothelium-dependent microvascular reactivity was shown to be impaired in acute sepsis in proportion to overall disease severity, and the degree of impairment at baseline predicted subsequent deterioration in organ function [84]. Studies in *Plasmodium falciparum* malaria have demonstrated endothelial dysfunction and decreased nitric oxide bioavailability with more marked changes in severe compared with mild malaria, and this work has led onto therapeutic intervention trials with L-arginine [85,86]. To date, results of clinical endothelial function tests have not been reported in dengue, but there are ongoing studies in Vietnam and Singapore. Preliminary results indicate that dysfunction occurs early, during the febrile phase, raising the possibility that this technique might be useful in predicting outcome. However, drawbacks include the need for a degree of patient cooperation, meaning that it is difficult to use the technique in younger children and unstable patients, and, at least at present, the cost of the disposable probes, which is likely to limit widespread application in resource-poor settings.

#### Monitoring flow in the microcirculation

Microcirculatory dysfunction causing tissue hypoperfusion is considered to play a major role in the pathophysiology of multiorgan failure in severe bacterial infections [87]. Early-onset multiorgan failure is thought to be mediated by inadequate tissue perfusion, whereas organ failure occurring later in the course of severe sepsis is likely to be related to mitochondrial failure and cell death [88]. In line with this hypothesis, haemodynamic optimization has been found to be beneficial in the early but not late stages of septic shock [89].

**Figure 2:**
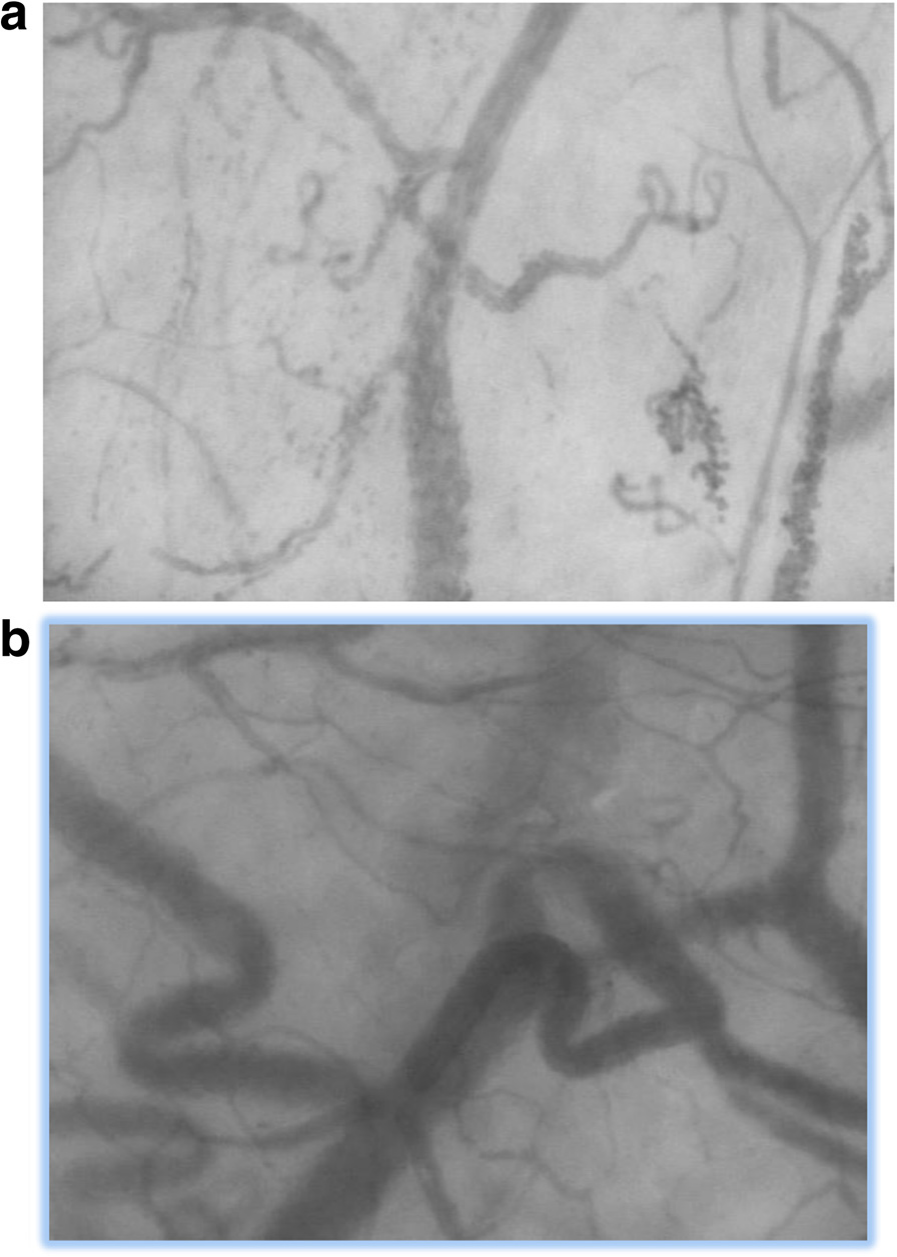
**Side-stream darkfield imaging (SDF) in dengue.** Example of still SDF images from **(a)** a patient with dengue in the late febrile phase, showing reduced total vessel density and perfused vessel density, plus extravasated red cells in the left upper and lower quadrants; **(b)** the same patient in the recovery phase with normal vessel density, flow rate and perfusion indices.

In the past two decades, bedside real-time imaging of the microcirculation has become possible with videomicroscopy using orthogonal polarisation spectral imaging (OPS) and side-stream darkfield imaging (SDF) [90]. It has been postulated that during the earliest phase of incipient shock, impaired microcirculatory flow and tissue hypoperfusion can be detected prior to the onset of overt cardiovascular collapse [91]. A decrease in the number of perfused capillaries, together with reduced flow rates and heterogeneity of the microcirculation, is associated with increased severity of both sepsis and malaria, and these changes are strong predictors of outcome [91,92]. Microcirculatory changes occur early in the course of the disease and are independent of arterial blood pressure, and their persistence is associated with mortality [91,93]. At present, alterations to microcirculatory flow patterns in dengue are largely unknown. In one case study from Brazil involving two patients with DSS, very abnormal microcirculatory patterns were identified, which improved following resolution of shock [94]. Our group in Vietnam is currently investigating the microcirculation using SDF technology in a large study of patients with suspected dengue, carrying out serial examinations from the early febrile phase throughout the evolution of the illness to see if the technique has prognostic value (Figure 2).

#### Monitoring intravascular volume status

The ability to detect and measure changes in intravascular volume early in the evolution of dengue, particularly during the compensated phase of shock, would be of real benefit for patient management. The compensatory reserve index (CRI) is a new computational algorithm that uses feature analysis of the arterial pulse waveform to track real-time changes in central blood volume [95]. Arterial waveform data are processed by the algorithm to calculate a CRI value between 0 and 1, where 1 represents normovolaemia and 0 represents the point of decompensation [96]. The CRI has been shown to correlate with estimated volume changes in a human disease model that simulates hypovolaemia due to haemorrhage, and to detect changes that occur during the compensatory phase of shock, preceding changes in conventional haemodynamic parameters. In addition, the index has been shown to distinguish between individuals with high and low tolerance to intravascular volume loss, and may therefore be helpful in identifying patients who are more vulnerable to shock. One advantage of this technique is that the CRI can be obtained from any standard monitor that generates a pulse waveform, including a finger pulse oximeter, and it is therefore completely non-invasive and relatively inexpensive. Studies to assess the utility of the CRI in predicting likely progression to shock, and to track fluid resuscitation status in patients with established shock, are ongoing in Southeast Asia.

Another non-invasive bedside technique with potential for monitoring intravascular volume is portable echocardiography [97]. Echocardiograms have the additional benefit of providing a functional cardiac assessment as well as an estimate of intravascular volume status, and are increasingly being used for haemodynamic assessment in patients with septic shock [98]. Cardiac involvement is now recognised as a feature of dengue [99], particularly in severe cases where myocardial impairment probably acts in concert with hypovolaemia to compound haemodynamic instability [100]. In such cases, assessing collapsibility of the inferior vena cava may be useful in predicting which patients are likely to be fluid-responsive. Although this method has been used mainly for ventilated patients [101], evidence is emerging that it may also be useful in spontaneously breathing patients [102]. Cardiac dysfunction probably also plays a role in less severe dengue disease. In one paediatric study, reduced cardiac output during the febrile phase was identified as being related both to reduced preload and to left ventricular impairment, with no apparent change in blood pressure [103]. As portable devices become increasingly accessible worldwide, inclusion of an echocardiographic assessment to assist with dengue risk prediction is likely to become a feasible option in many mid-level healthcare facilities.

## Conclusion

Predicting outcome in dengue remains challenging, and the search for more robust methods continues. Although warning signs are considered a key component for early recognition of potentially severe disease, the current evidence for any particular clinical or laboratory marker is weak. A major global study currently in progress, aiming to recruit 10,000 to 12,000 suspected dengue cases during the early febrile phase, should provide useful information for risk prediction(ClinicalTrials.govID:NCT01550016). However, given the rather broad range of signs and symptoms seen in dengue, inclusion of one or more specific biomarkers is likely to be needed in order to develop a robust algorithm. A number of viral, immunological and endothelial biomarkers have been proposed from small studies, and there is hope that, if these arevalidated in a large patient cohort, algorithms incorporating such biomarkers might prove sufficiently sensitive and specific to be clinically useful, particularly in endemic areas where the case burden is high. However, as the vascular system is the essential target in severe dengue, alternative approaches that focus primarily on the clinical vascular function tests described above could also prove to be helpful. Although these are relatively expensive and limited to centres with specialist expertise at present, if these are shown to be reliable, their non-invasive nature makes them an attractive option, especially for repeated assessments on children, and costs are likely to come down if the techniques are widely deployed. Finally, ongoing research efforts directed towards identifying robust early predictors for severe disease are crucial to ensure that if effective antiviral or disease-modifying drugs do become available in the future, these agents can be used to maximal advantage for the patients most at risk.
